# Interindividual differences in dyspnea during acute hypoxic exercise in healthy adults: A single exposure to simulated altitude at ~9000 ft

**DOI:** 10.14814/phy2.71085

**Published:** 2026-08-30

**Authors:** Jacob L. Schwartz, Aisha Khan, Robert F. Bentley

**Affiliations:** ^1^ Faculty of Kinesiology and Physical Education University of Toronto Toronto Ontario Canada

**Keywords:** autonomic activity, cycling, microvascular responsiveness, muscle oxygenation, normobaric hypoxia

## Abstract

The growing accessibility of simulated altitude facilities (~13%–16% fraction of inspired oxygen [FIO_2_]) has expanded their use to broader recreational populations. Although acute normobaric hypoxia reduces arterial oxygen saturation and increases ventilatory and sympathetic responses, interindividual variability in dyspnea during hypoxic exercise remains poorly characterized. Twenty‐two healthy individuals (24 ± 4 years, 50% female) completed one 90‐min exposure at an altitude facility (FIO_2_ ≈ 15 ± 0.1%) that included an assessment of heart rate variability (autonomic activity), a vascular occlusion test (microvascular responsiveness), and submaximal cycling at 60% heart rate reserve. *Vastus lateralis* (*VL*) oxygen saturation and heart rate were measured throughout. Marked interindividual variability in dyspnea derived from the Multidimensional Dyspnea Profile was observed across the cycling protocol. While resting autonomic activity (all *p* > 0.326) and microvascular responsiveness (all *p* > 0.138) were not associated with dyspnea perception, greater *VL* deoxygenation was strongly correlated with dyspnea during cycling (all *r* > −0.558, all *p* < 0.007) with the interpretation unchanged when adjusted for wattage. Additionally, males exhibited higher dyspnea (*p* = 0.022) and greater *VL* deoxygenation (−32 ± 14% vs. −15 ± 18%, *p* = 0.022) compared to females. Combined, these findings highlight the importance of interindividual variability in dyspnea during hypoxic exercise and warrant further investigation into its functional implications.

## 1 INTRODUCTION

Simulated altitude (i.e., normobaric hypoxia) challenges oxygen delivery by reducing the fraction of inspired oxygen (FIO_2_), thereby lowering arterial oxygen pulse saturation (SpO_2_) and limiting oxygen availability to active skeletal muscle (Amann et al., 2007; Hogan et al., 1999). Reduced SpO_2_ during normobaric hypoxia stimulates carotid body chemoreceptors, leading to increased ventilatory drive and sympathetic activation in an effort to maintain systemic oxygen delivery (Somers et al., 1989a, 1989b). These compensatory responses may also contribute to the sensation of dyspnea (Takano et al., 1997), defined as the subjective experience of breathing discomfort (Parshall et al., 2012). However, substantial individual variability in dyspnea perception during acute hypoxic exercise has been observed anecdotally by our research team, aligning with dyspnea evidence during normoxic exercise (Takano et al., 1997) alongside resting dyspnea perceptions following 30‐min of normobaric hypoxia at FIO_2_ 12%, 14.5%, and 17% (Costello et al., 2020). Together, these findings suggest that dyspnea may be particularly heterogeneous under hypoxic conditions, and understanding this variability is important, as the growing accessibility of normobaric hypoxic facilities (typically ~13%–16% FIO_2_) has expanded their use to broader recreational and non‐athletic populations (Girard et al., 2020; Liu et al., 2025) beyond the traditional confines of endurance athletes (Flaherty et al., 2016). Indeed, the hypoxic ventilatory response arising from hypoxic stimulation of peripheral chemoreceptors may vary considerably both within and between individuals, thereby contributing to interindividual differences in arterial oxygenation during hypoxic exposure (Teppema & Dahan, 2010). Consequently, variability in the hypoxic ventilatory response may contribute to heterogeneity in physiological and perceptual responses, including the sensation of dyspnea.

Oxygen delivery to active skeletal muscle is determined by both arterial oxygen content and muscle blood flow, the latter of which is controlled by resistance vessels within the microvasculature (Delp & Laughlin, 1998). Given the reductions in arterial oxygen content with simulated altitude exposure, muscle blood flow, and by extension microvascular responsiveness, is an important contributor to oxygen delivery–demand matching. Martin et al. (2013) demonstrated reduced microvascular reactivity in acclimatizing healthy volunteers at high altitude (4900 and 5600 m), with considerable interindividual variability in these responses (Martin et al., 2013), suggesting that such heterogeneity may contribute to differences in oxygen delivery–demand matching. Impaired matching may increase local red blood cell desaturation (Schwartz et al., 2025) and reliance on nonoxidative metabolism, promoting the accumulation of metabolic byproducts that activate group III/IV muscle afferents, thereby augmenting ventilatory responses and contributing to dyspnea (Amann et al., 2010). Moreover, autonomic regulation may represent an additional mechanism contributing to interindividual dyspnea responses during hypoxic exercise. Botek et al. (2015) reported that exposure to acute normobaric hypoxia (FIO_2_ ≈ 9.6%) resulted in interindividual SpO_2_ responses, with greater desaturation associated with a shift toward sympathetic dominance and reduced vagal activity (Botek et al., 2015). Similarly, Taralov et al. (2015) reported that lower SpO_2_ was associated with reduced autonomic activity during acute normobaric hypoxia (FIO_2_ ≈ 12.3%) (Taralov et al., 2015). Given that reductions in SpO_2_ stimulate peripheral chemoreceptors and increase ventilatory drive (Somers et al., 1989a, 1989b), interindividual differences in autonomic regulation may help explain the observed variability in dyspnea responses. Combined, associations between peripheral physiological factors beyond ventilatory control, such as microvascular responsiveness and autonomic activity, and the noted anecdotal variability in individual dyspnea perception during acute hypoxic exercise remain underexplored.

Biological sex may also contribute to dyspnea perception during hypoxic exposure, as females tend to exhibit greater parasympathetic modulation (Koenig & Thayer, 2016; Sztajzel et al., 2008), lower sympathetic vasoconstrictor responsiveness, and reduced vascular resistance during sympathetic stimulation (Hogarth et al., 2007), which may, under hypoxic stress, facilitate oxygen delivery‐demand matching. Accordingly, the comparatively lower parasympathetic modulation and greater sympathetic vasoconstrictor responsiveness in males may impair oxygen delivery‐demand matching during hypoxic exercise, potentially contributing to greater skeletal muscle deoxygenation, ventilatory drive, and perceived dyspnea.

To our knowledge, no study has simultaneously examined associations between microvascular responsiveness, autonomic activity, and perceived dyspnea during acute hypoxic exercise, nor assessed local skeletal muscle oxygenation alongside these measures. Therefore, the purpose of this study was to investigate how resting microvascular responsiveness and autonomic activity relate to physiological and perceptual responses during submaximal cycling under normobaric hypoxia (FIO_2_ ≈ 15%, equivalent to ~9000 ft). We hypothesized that interindividual variability in dyspnea perception would be observed, and that individuals with greater dyspnea perception would exhibit (1) attenuated microvascular responsiveness, (2) greater sympathetic activity, and (3) greater skeletal muscle deoxygenation during cycling. We further hypothesized that males would report greater dyspnea than females.

## 2 MATERIALS AND METHODS

### 2.1 Participants and sample size estimation

Twenty‐two healthy individuals participated in this study (age: 24 ± 4 years, 50% female). Participants were recruited through posters displayed on campus at the University of Toronto (Toronto, ON, Canada) and study advertisements posted on the website of the Faculty of Kinesiology & Physical Education. The University of Toronto Health Sciences research ethics board approved this study (Approval No. 47308) according to the terms of the Declaration of Helsinki and all participants provided written informed consent before their participation. Females were tested during the follicular phase of their menstrual cycle. Participants were included if they (1) were between the ages of 18 and 35 years, (2) had a body mass index between 18.5 and 29.9 kg/m^2^, (3) self‐reported as healthy with successful completion of a Get Active Questionnaire (GAQ), and (4) had no past history of diagnosis of COVID‐19 that required hospitalization. Participants were excluded if they (1) were unable to perform cycling exercise, (2) regularly used tobacco, (3) had a skinfold thickness above the right *vastus lateralis* >12.5 mm, (4) had any prior history of cardiovascular or respiratory disease, or (5) had prior altitude exposure, operationally defined as history of training under hypoxic conditions but excluding travel for pleasure and tourist related activities.

Given the limited availability of data examining the relationship between perceptual and physiological variables during hypoxic exercise, sample size estimation was informed by preliminary pilot data (*n* = 10). This pilot demonstrated a strong correlation (*r* = 0.64) between exertional dyspnea and skeletal muscle oxygen saturation (SmO_2_); a measure reflecting local muscle oxygen delivery and extraction/utilization during exercise. Based on a two‐tailed correlation analysis with *α* = 0.05 and 80% power, 17 participants were required.

### 2.2 Experimental design

**FIGURE 1:**
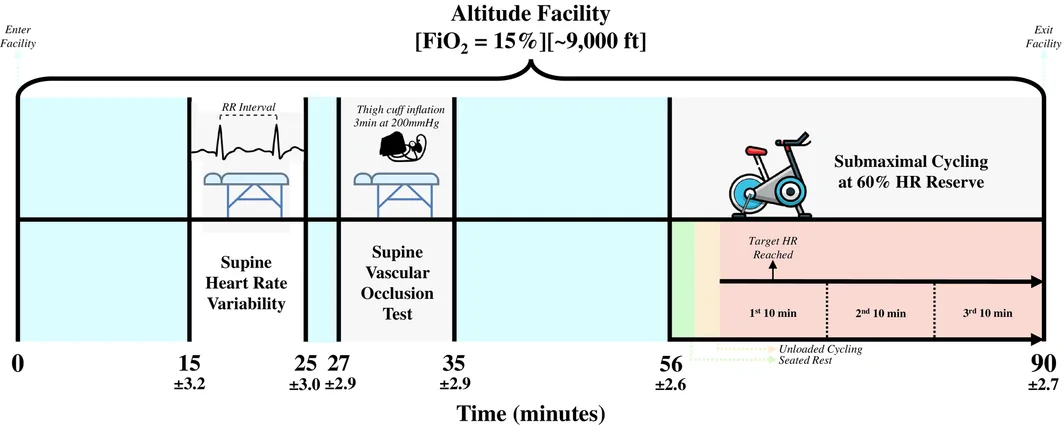
Visual schematic of the experimental visit. Participants completed a 90‐min testing session in a simulated altitude facility under normobaric hypoxic conditions (FIO_2_ ≈ 15 ± 0.1%, ~9000 ft). The protocol included supine heart rate variability, a vascular occlusion test, and submaximal cycling at 60% heart rate (HR) reserve. Heart rate and *vastus lateralis* muscle oxygen saturation were measured continuously.

This study employed a single‐group, cross‐sectional design. Participants completed one 90‐min testing session in a simulated altitude facility (~1200 sq. ft. private altitude gym in Toronto, ON, Canada) that included an assessment of heart rate variability (HRV), a vascular occlusion test (VOT), and submaximal cycling (Figure 1). All assessments were performed under normobaric hypoxic conditions (FIO_2_ ≈ 15 ± 0.1%, equivalent to ~9000 ft. with continuous air exchange). Participants were scheduled for data collection between 9 am and 3:30 pm. Participants were screened to ensure they met all inclusion and exclusion criteria prior to their study visit. During screening, standard anthropometric measurements were obtained, including biological sex, age, height, weight, and skinfold thickness of the right vastus lateralis ~14 cm above the patella. Participants were instructed to refrain from exercise for 24 h, from alcohol, caffeine, and energy‐altering substances for 12 h, and from food for 4 h prior to data collection. A seven‐day physical activity questionnaire adapted from Sarkin et al. (Sarkin et al., 1997) was completed to confirm current physical activity habits. Perceived breathlessness was evaluated using the Medical Research Council (MRC) Breathlessness Questionnaire, which grades dyspnea severity on a five‐point scale, with higher scores indicating greater perceived limitation (Bestall et al., 1999). Prior to entering the altitude facility, all participants received identical standardized verbal and written information about the experimental procedures without discussing the expected magnitude or direction of dyspnea responses.

### 2.3 Heart rate variability

The HRV assessment commenced ~15 min into hypoxic exposure. Participants lay quietly in a supine position on a portable testing bed for a total of 15 min. Data from the initial 10 min were excluded from analysis and the subsequent 5 min epoch was assessed to allow participants to achieve a resting physiological state (Task Force, 1996).

### 2.4 Vascular occlusion test

The VOT commenced ~2 min following the HRV assessment. Participants remained supine on a portable testing bed while a thigh blood pressure cuff (Amico Group; Aurora, ON, Canada) was positioned proximally on the upper right thigh, ensuring adequate space distal to the cuff for near‐infrared spectroscopy probe placement (Moxy NIRS; Fortiori Design LLC, Hutchinson, MN, USA). Measurements were recorded during a 2‐min baseline period, a 3‐min occlusion period (Iannetta et al., 2019), and a 3‐min recovery period. The cuff was inflated to 200 mmHg to achieve arterial occlusion and was removed at the end of the occlusion phase to allow for an immediate and unrestricted reperfusion response.

### 2.5 Submaximal cycling exercise

The cycling protocol commenced ~56 min into hypoxic exposure. Participants first completed 2 min of seated rest and then 2 min of unloaded cycling. Following this, participants performed 30 min of standardized, moderate intensity submaximal cycling at 60% of their heart rate reserve (HRR)–a common training zone amongst the recreationally active population. Target heart rate (HR) was calculated as:

$$\text{Target}\;\mathrm{HR}=\left({\mathrm{HR}}_{\max}-{\mathrm{HR}}_{\text{rest}}\right)\times 0.60+{\mathrm{HR}}_{\text{rest}}$$

where maximum heart rate (${\mathrm{HR}}_{\max}$) was estimated as 220 minus the participant's age in years, and resting heart rate (HR_rest_) was derived from the final minute of the HRV assessment, during which values were confirmed to be stationary. The cycle ergometer (Technogym Skillbike, Technogym S.p.A., Cesena, Italy) was wirelessly connected to a Frontier X2 (Fourth Frontier Technologies LLC, Austin, USA) heart rate monitor, and automatically adjusted workload to maintain each participant's target HR throughout the session. Participants reached their target HR within the first 5 min and pedaling cadence was self‐selected within a range of 60–80 revolutions per minute (rpm) and subsequently maintained throughout cycling. Cycling wattage (W) was continuously recorded via the Technogym Skillbike using the manufacturer's integrated application at a sampling frequency of 1 Hz and subsequently averaged into 30‐s time bins. Seat height was individually adjusted to ensure comfort and a consistent cycling posture, defined as a slight knee bend at full pedal extension. Participants were blinded to elapsed time throughout the simulated altitude exposure and completed assessments.

### 2.6 Heart rate, heart strain, and arterial saturation

Participants were fitted with two wireless HR monitors: a Polar V800 (Polar Electro Oy, Kempele, Finland) and a Frontier X2. Sensors were secured on the chest just below the sternum using elastic straps, with the Polar device positioned inferior to the Frontier to ensure consistent placement across participants. R–R interval data for HRV were obtained from the Polar V800, while heart rate was recorded continuously using the Frontier X2, from which heart strain, a representation of ST segment deviation, was derived for each physiological assessment. Strain was interpreted as insignificant (<0.1 mV), moderate (0.1–0.2 mV) or high (>0.2 mV) as per manufacturer. SpO_2_ was measured continuously via pulse oximetry (ChoiceMMed, Beijing Choice Electronic Technology Co., Ltd., Beijing, China) on the index finger of the right hand. SpO_2_ values were monitored to confirm stable arterial oxygen saturation throughout the hypoxic exposure.

### 2.7 Skeletal muscle oxygen saturation

Skeletal muscle oxygen saturation (SmO_2_) of the right *vastus lateralis* was measured by continuous wave near‐infrared spectroscopy (Moxy, NIRS) at 2 Hz. This device employs four wavelengths of near‐infrared light (680, 720, 760, and 800 nm) with a penetration depth of 12.5 mm based upon light detector and emitter distances and provides relative changes in the proportional concentrations of oxygenated and deoxygenated hemoglobin (Hb)/myoglobin (Mb) (SmO_2_ = oxy‐[Hb/Mb]/total [Hb/Mb], %). Prior to altitude exposure, the device was secured to the skin over the *vastus lateralis* ~14 cm above the patella with adhesive tape and covered with a light‐blocking elastic bandage. Correct device placement was confirmed when the quadriceps was voluntarily contracted, and the presence of desaturation was identified. Placement was modified and reconfirmation was completed if needed. SmO_2_ was measured continuously during the hypoxic exposure.

### 2.8 Ratings of perceived exertion and dyspnea assessment

Within the final 30 s of minutes 10, 20, and 30 of cycling, whole‐body rating of perceived exertion (RPE) was assessed using the Borg 6–20 scale. Prior to exercise, participants were oriented to the scale and read a script modified from Faulkner & Eston (Faulkner & Eston, 2007). During the same time period, participants also completed the Multidimensional Dyspnea Profile, which assesses overall breathing discomfort, sensory qualities of breathlessness, and associated emotional responses (Banzett et al., 2015). Before exercise, participants were read the standardized Multidimensional Dyspnea Profile instructions and given the opportunity to clarify any questions to ensure accurate and consistent responses across participants.

### 2.9 Data analysis

#### 2.9.1 Heart rate variability

Prior to analysis, individual R–R intervals were processed and abnormal intervals were removed in accordance with the methods outlined by Kemper et al. (Kemper et al., 2007) and replaced using cubic spline interpolation. Processed R–R interval data were imported into Kubios HRV software (version 4.1.2; Kubios HRV, Kuopio, Finland) for analysis. Welch's periodogram was applied using a 256‐s window with 50% overlap. Low‐frequency (LF) spectral bands were defined as 0.04–0.15 Hz, and high‐frequency (HF) spectral bands were defined as 0.15–0.40 Hz. R–R interval data were interpolated at 4 Hz. No artifact correction was applied within Kubios HRV, and respiration rate was not controlled. Variables were log‐transformed when the assumption of normality was violated.

#### 2.9.2 Vascular occlusion test

All oxygenation data are a delta (∆) from the average of the last 30 s of the 2‐min baseline period and placed into one second time bins. Desaturation rate (%· s^−1^) was quantified as the 180 s linear downslope following complete arterial occlusion until cuff deflation and removal. The nadir (%) was quantified as the lowest SmO_2_ value achieved during arterial occlusion. Reoxygenation rate (%· s^−1^) was quantified as the 10 s linear upslope immediately following the nadir value. Hypersaturation area under the curve (AUC) was quantified as the 2‐min AUC following return to baseline (i.e., from the first positive SmO_2_ value). Only positive ΔSmO_2_ values were included in the AUC calculation to quantify the magnitude of the hypersaturation response above baseline. In participants that did not display a hypersaturation response following cuff deflation and removal, an AUC value of zero was assigned (*n* = 1). The total magnitude of change was quantified as the difference between the peak and nadir SmO_2_. NIRS‐derived VOT parameters were used as established surrogate indices of local microvascular responsiveness (i.e., vascular reactivity at the microcirculatory level), reflecting local tissue oxygenation kinetics during ischemia and reperfusion (Kriel et al., 2024; Rasica et al., 2022). The changes in skeletal muscle oxygen saturation relative to the pre‐occlusion baseline reflect vascular responsiveness to ischemia‐induced metabolite accumulation. The NIRS‐derived VOT therefore provides an assessment of local skeletal muscle oxygenation kinetics during occlusion and reperfusion, but does not provide a direct measure of microvascular blood flow, capillary hemodynamics, or vascular conductance.

#### 2.9.3 Submaximal cycling exercise

SmO_2_ and HR data were averaged over 30‐s time bins. The final minute of rest, unloaded cycling, and each 10‐min cycling window were used for statistical analysis. AUC analysis derived from 30‐s time bins was also performed for all variables across the entire cycling duration to capture the overall magnitude of the physiological response.

### 2.10 Dyspnea group classification

Participants were categorized into high and low dyspnea groups using tertiles based on dyspnea scores at 30 min of cycling. Dyspnea scores were derived from the immediate perception domain of the Multidimensional Dyspnea Profile. Scores were rank‐ordered, and the lower tertile (*n* = 7) and higher tertile (*n* = 7) dyspnea groups were analyzed while the middle tertile was not. This exploratory approach was applied post hoc to facilitate comparisons between individuals with distinct dyspnea responses to the Multidimensional Dyspnea Profile assessment.

### 2.11 Statistical analysis

Statistical analysis was completed using a combination of JASP (JASP Team, v. 0.17.2) and SigmaPlot 12.0 (Systat Software, Inc.). Normality of data was assessed with a Shapiro–Wilk test. Parametric data are presented as means ± SD. Nonparametric data are presented as median, interquartile range (Q1–Q3). A one‐way repeated‐measures analysis of variance (RMANOVA) was conducted to assess the overall effect of time (10, 20, and 30 min of cycling). Separate mixed‐model RMANOVAs were then performed to examine the effect of time with sex (male vs. female) or dyspnea group (high vs. low) as between‐subject factors, including time × sex and time × dyspnea interactions respectively. Following significant F‐statistics, Bonferroni corrected post hoc *t*‐tests were completed. Assumptions of normality and homogeneity of variance in the RMANOVAs were met. When the assumption of sphericity was violated, a Greenhouse–Geisser correction was applied while the robustness of the F‐statistic was relied on when the assumption of normality was not met (Blanca et al., 2023). Analyses involving tertile‐based dyspnea classification were considered exploratory and were interpreted accordingly.

For participant characteristics, individuals with high dyspnea scores were compared with individuals with low dyspnea scores using independent *t*‐tests or Mann–Whitney *U* tests as appropriate. In addition, males were compared with females using independent *t*‐tests or Mann–Whitney *U* tests. To determine the proportion of males/females presenting with high versus low dyspnea, a Fisher's exact test was computed. Furthermore, Pearson or Spearman correlational analyses were performed, as appropriate, between dyspnea scores and physiological variables. Associations between SmO_2_ and dyspnea were further assessed with a sensitivity analysis using Huber robust regression. Huber regression down‐weights potential influential observations with larger residuals rather than excluding them, allowing for assessment of whether the observed associations remained robust to potentially influential observations. Interpretation of significant correlations was determined according to weak, moderate, and strong (Akoglu, 2018). For SmO_2_ adjusted for cycling wattage, Pearson correlations with bootstrapped (1000 simulations) confidence intervals were used to provide robust estimates under non‐normality. Spearman rank correlations were also computed as a non‐parametric sensitivity analysis to assess consistency in the direction of associations. LF, HF, and the LF/HF ratio were log‐transformed prior to analysis to satisfy normality assumptions; non‐transformed values are presented for interpretability. Cycling data were expressed as Δ from resting baseline to highlight the physiological effects of exercise. Statistical significance was set at *p* < 0.05 for all analyses.

## 3 RESULTS

### 3.1 Participant characteristics and interindividual differences in dyspnea during cycling

**FIGURE 2:**
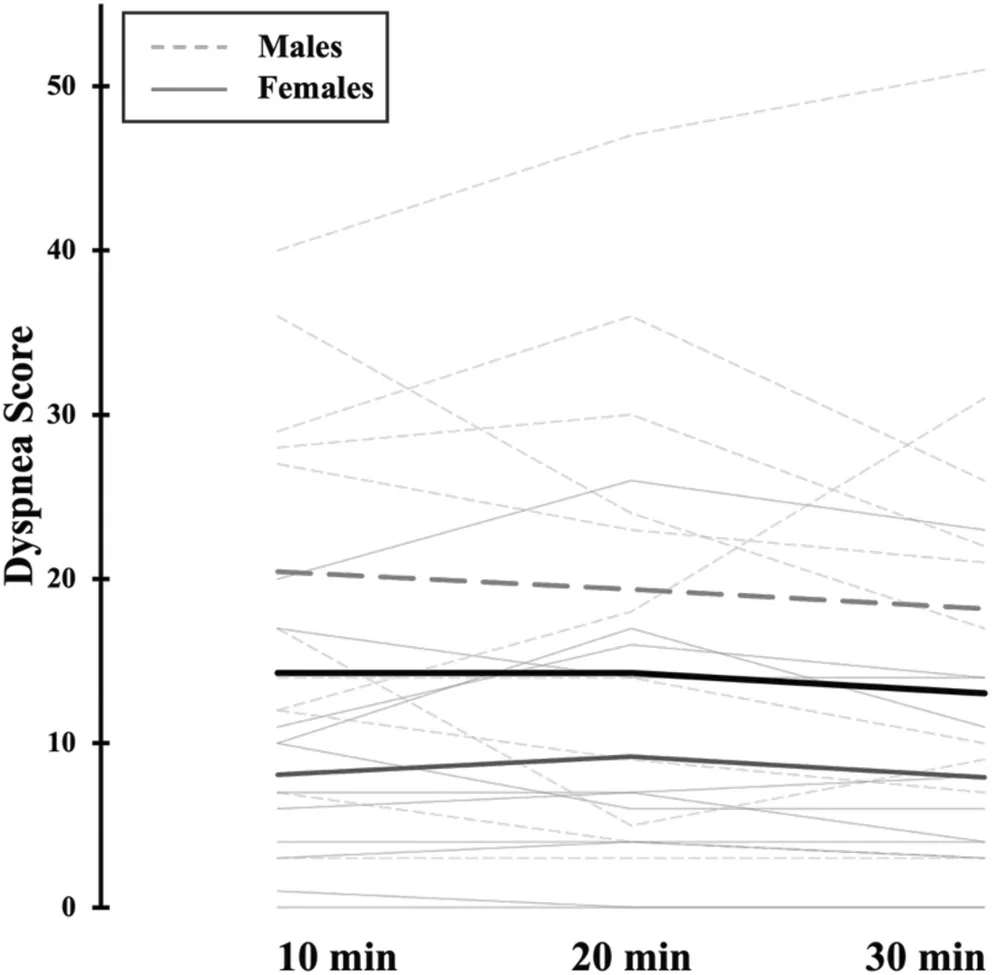
Interindividual variability in dyspnea during submaximal cycling. Dyspnea (immediate perception domain of the Multidimensional Dyspnea Profile; sum of sensory qualities and breathing discomfort) was assessed at 10, 20 and 30 min during cycling at 60% heart rate reserve under normobaric hypoxic conditions (FIO_2_ ≈ 15 ± 0.1%, ~9000 ft). Thin lines represent individual participants (*n* = 22). Females are shown as solid lines and males as dashed lines. Sex‐specific means are shown as bold gray lines, and the overall mean is shown in black.

Participant characteristics are presented in Table 1. Males were taller (*p* < 0.001) and had lower adipose tissue thickness at the *vastus lateralis* (*p* < 0.001) compared to females. Marked interindividual variability in dyspnea responses derived from the immediate perception domain was observed across the 30‐min submaximal cycling protocol (Figure 2). While the group mean remained relatively stable over time (*p* = 0.492), individual trajectories varied considerably, with some participants demonstrating increases, decreases, or stable responses across the protocol. High versus low dyspnea groups did not differ with respect to anthropometry or physical activity levels (all *p* > 0.195). The proportion of males was non‐significantly higher in the high dyspnea group compared to the low dyspnea group (86 vs. 29%, *p* = 0.103). SpO_2_ and heart strain during supine rest did not differ between dyspnea groups or between sexes (all *p* > 0.144). Similarly, time spent in the simulated altitude facility did not differ between dyspnea groups or between sexes (both *p* > 0.390). No significant associations were observed between time of testing and dyspnea throughout cycling (all *p* > 0.356).

**TABLE 1:** Participant characteristics.

Variable	All (*n* = 22)	High dyspnea (*n* = 7)	Low dyspnea (*n* = 7)	*p*‐value	Male (*n* = 11)	Female (*n* = 11)	*p*‐value
Sex (% male)	50	86	29	0.103	‐	‐	‐
Age (years)	24 ± 4	23 ± 3	26 ± 3	0.195	24 ± 4	24 ± 4	0.955
Height (cm)	172 ± 8	174 ± 8	169 ± 8	0.232	177 ± 8	167 ± 4	**<0.001** ^a^
Weight (kg)	69 ± 11	71 ± 13	64 ± 7	0.237	73 ± 13	66 ± 7	0.115
BMI (kg/m^2^)	23.4 ± 3.0	23.4 ± 4.1	22.4 ± 2.0	0.572	23.2 ± 3.3	23.7 ± 2.7	0.708
Adipose tissue thickness (mm)	6.2 ± 2.5	5.3 ± 1.9	6.7 ± 3.3	0.338	4.3 ± 1.8	8.1 ± 1.4	**<0.001** ^a^
7‐day PAR score (METs/week)	268 ± 35	263 ± 22	265 ± 20	0.902	257 ± 16	279 ± 46	0.365
MRCBQ grade	0 (0–0)	0 (0–1)	0 (0–0)	‐	0 (0–1)	0 (0–0)	0.303
Supine rest SpO_2_ (%)	94 ± 2	94 ± 2	92 ± 2	0.144	94 ± 2	94 ± 2	1.000
Supine rest heart strain (mV)	0.038 ± 0.032	0.030 ± 0.017	0.044 ± 0.044	0.436	0.039 ± 0.029	0.036 ± 0.036	0.691
Cycling heart strain (mV)	0.029 ± 0.018	0.014 ± 0.013	0.031 ± 0.013	**0.031**	0.027 ± 0.022	0.030 ± 0.013	0.733
Total hypoxic exposure (min)	90 ± 3	91 ± 3	89 ± 3	0.390	90 ± 3	90 ± 3	0.530

*Note*: Data are presented as mean ± SD or median (Q1–Q3). High and low dyspnea groups were defined using tertiles based on 30‐min dyspnea scores (immediate perception domain of the Multidimensional Dyspnea Profile), with the upper and lower tertiles retained and the middle tertile excluded.

Abbreviations: BMI, body mass index; MET, metabolic equivalent; MRCBQ, medical research council breathlessness questionnaire; PAR, physical activity recall; SpO_2_, arterial oxygen pulse saturation.

^a^ Statistically significant difference between males and females (*p* < 0.05). Significant *p*‐values have been bolded.

The immediate perception domain of dyspnea represents the summed contribution of breathing discomfort (A1) and the five sensory qualities (SQ1–SQ5) (Table 2). Higher scores were observed for each individual component and for the overall immediate perception domain in the high dyspnea group (all *p* < 0.001). Separately, males reported elevations in select sensory qualities, including air hunger (SQ2; *p* = 0.028), mental breathing effort (SQ4; *p* = 0.010), and hyperpnea (SQ5; *p* = 0.002), alongside the overall immediate perception domain (*p* = 0.022).

**TABLE 2:** Multidimensional dyspnea profile.

Variable	All (*n* = 22)	High dyspnea (*n* = 7)	Low dyspnea (*n* = 7)^a^	Male (*n* = 11)	Female (*n* = 11)
*10 min*					
Breathing discomfort (A1)	3 ± 2	5 ± 1	1 ± 1	4 ± 2	2 ± 1
Physical breathing effort (SQ1)	3 (1–4)	5 (4–6)	1 (0–2)	3 (2–5)	2 (1–3)
Air hunger (SQ2)^b^	1 (0–3)	5 (3–7)	0 (0–0)	3 (0–6)	0 (0–2)
Tightness (SQ3)	1 (0–2)	4 (3–5)	0 (0–1)	1 (0–5)	0 (0–1)
Mental breathing effort (SQ4)^b^	2 (0–3)	3 (3–5)	0 (0–0)	3 (1–4)	1 (0–2)
Hyperpnea (SQ5)^b^	3 (2–5)	6 (5–7)	1 (0–2)	5 (4–7)	2 (1–3)
Immediate perception domain^b^	12 (6–19)	28 (24–33)	3 (2–5)	17 (12–29)	7 (4–11)
*20 min*					
Breathing discomfort (A1)	3 ± 2	5 ± 2	1 ± 1	3 ± 2	2 ± 2
Physical breathing effort (SQ1)	2 (1–4)	5 (4–6)	1 (0–2)	3 (1–5)	2 (1–3)
Air hunger (SQ2)^b^	1 (0–4)	4 (4–6)	0 (0–0)	3 (1–5)	1 (0–2)
Tightness (SQ3)	1 (0–2)	4 (3–5)	0 (0–0)	1 (0–4)	0 (0–1)
Mental breathing effort (SQ4)^b^	2 (0–3)	4 (4–8)	0 (0–0)	3 (2–6)	1 (0–2)
Hyperpnea (SQ5)^b^	3 (1–5)	5 (5–6)	1 (0–1)	5 (3–5)	2 (1–3)
Immediate perception domain^b^	12 (4–22)	26 (24–33)	4 (2–4)	18 (7–27)	7 (4–15)
*30 min*					
Breathing discomfort (A1)	3 ± 2	5 ± 2	1 ± 1	3 ± 2	2 ± 2
Physical breathing effort (SQ1)	2 (1–3)	5 (4–6)	1 (1–1)	3 (1–5)	2 (1–3)
Air hunger (SQ2)^b^	1 (0–3)	3 (3–4)	0 (0–0)	2 (1–3)	0 (0–2)
Tightness (SQ3)	1 (0–2)	4 (2–4)	0 (0–0)	1 (0–3)	0 (0–2)
Mental breathing effort (SQ4)^b^	2 (0–2)	3 (3–6)	0 (0–0)	2 (2–4)	0 (0–2)
Hyperpnea (SQ5)^b^	2 (1–4)	6 (4–8)	1 (0–1)	4 (2–7)	1 (1–2)
Immediate perception domain^b^	10 (4–20)	23 (22–29)	3 (2–4)	17 (8–24)	6 (4–13)

*Note*: Parametric variables are presented as mean ± SD. Non‐parametric variables are presented as median (Q1‐Q3). A1, breathing discomfort (immediate unpleasantness); SQ1–SQ5, sensory qualities. All items were rated on a 0–10 scale. The immediate perception domain represents the sum of A1 and SQ1–SQ5 scores. Higher scores indicate greater perceived dyspnea. The emotional response domain (A2) was not reported due to minimal variability, as the majority of responses were zero on the 0–10 scale. No significant interactions were observed.

^a^ Significant main effect of dyspnea group (high vs. low) was observed for all variables (*p* < 0.05).

^b^ Significant main effect of sex was observed for SQ2, SQ4, SQ5, and the immediate perception domain (*p* < 0.05).

### 3.2 Describing the intensity of submaximal cycling

Descriptors of submaximal cycling intensity are presented in Table 3. Cycling was completed at a target of 60% HRR and did not differ across exercise time points (*p* = 0.730) or between dyspnea groups (*p* = 0.292). A significant time × sex interaction was observed for heart rate (*p* = 0.031); however, Bonferroni‐adjusted post hoc comparisons revealed no significant pairwise differences (all *p* > 0.857). While heart strain during cycling did not differ between males and females (*p* = 0.733), it was greater in the low dyspnea group compared to the high dyspnea group (0.031 ± 0.013 vs. 0.014 ± 0.013 mV, *p* = 0.031; Table 1). There were no main effects or interactions for SpO_2_ (all *p* > 0.081). Wattage was lower at 20 min compared to 10 min (*p* < 0.001) but did not differ between 20 and 30 min (*p* = 0.246). Wattage also did not differ by dyspnea group (*p* = 0.920) nor sex (*p* = 0.110), and no interactions were observed (all *p* > 0.328). Finally, rating of perceived exertion (RPE) was higher at 20 min than at 10 min (*p* = 0.006) while 20 and 30 min were not different (*p* = 0.257). RPE was higher in the high dyspnea group (*p* = 0.003), while males reporting a higher RPE than females at 10 min (13 ± 1 vs. 11 ± 1, *p* = 0.009), but not at 20 or 30 min (both *p* > 0.736).

**TABLE 3:** Submaximal cycling data.

Variable	All (*n* = 22)	High dyspnea (*n* = 7)	Low dyspnea (*n* = 7)	Male (*n* = 11)	Female (*n* = 11)
*10 min*					
HR (bpm)	142 ± 6	143 ± 6	139 ± 7	144 ± 6	140 ± 5
Wattage (W)	115 (93–163)	126 (102–171)	114 (90–181)	148 (108–186)	97 (90–130)
∆SmO_2_ (%)	−30 ± 17	−39 ± 16	−24 ± 16	−37 ± 15	−23 ± 16
SpO_2_ (%)	92 ± 3	93 ± 4	90 ± 2	93 ± 4	91 ± 2
RPE (Borg 6–20)	12 ± 2	13 ± 1	11 ± 1	13 ± 1	**11 ± 1** ^b^
*20 min*					
HR (bpm)	142 ± 5	141 ± 6	141 ± 6	141 ± 6	143 ± 4
Wattage (W)	**105 (88–154)** ^a^	114 (102–150)	96 (79–162)	125 (102–164)	90 (81–117)
∆SmO_2_ (%)	**−22 ± 21** ^a^	−33 ± 17	−19 ± 17	−31 ± 17	−12 ± 21
SpO_2_ (%)	91 ± 3	92 ± 3	90 ± 2	92 ± 3	91 ± 3
RPE (Borg 6–20)	**13 ± 2** ^a^	14 ± 1	11 ± 1	14 ± 2	12 ± 1
*30 min*					
HR (bpm)	141 ± 5	142 ± 5	137 ± 5	143 ± 5	140 ± 5
Wattage (W)	102 (84–143)	105 (95–133)	92 (78–155)	122 (95–167)	97 (82–115)
∆SmO_2_ (%)	−18 ± 18	−27 ± 14	−16 ± 16	−27 ± 14	−9 ± 18
SpO_2_ (%)	92 ± 3	92 ± 3	90 ± 2	92 ± 3	91 ± 3
RPE (Borg 6–20)	12 ± 2	13 ± 2	12 ± 1	13 ± 2	12 ± 1

*Note*: Parametric variables are presented as mean ± SD. Non‐parametric variables are presented as median (Q1–Q3). A main effect of dyspnea group was present for RPE. A main effect of sex was present for ∆SmO_2_ and RPE.

Abbreviations: HR, heart rate; RPE, rating of perceived exertion; SmO_2_, muscle oxygen saturation; SpO_2_, arterial oxygen pulse saturation.

^a^ Significantly different from the preceding time point based on post hoc comparisons (*p* < 0.05). Significant differences have been bolded.

^b^ Significant difference between males and females at the specified time point based on post hoc comparisons following a significant time × sex interaction (*p* < 0.05). Significant differences have been bolded.

### 3.3 Skeletal muscle oxygenation in relation to dyspnea

**FIGURE 3:**
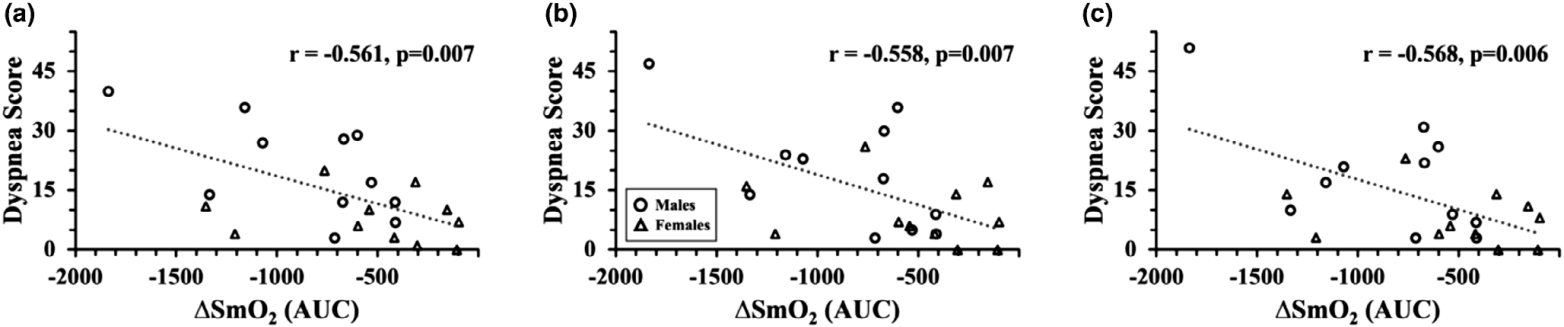
Relationship between dyspnea and muscle oxygen saturation. Dyspnea (immediate perception domain of the Multidimensional Dyspnea Profile; sum of sensory qualities and breathing discomfort) at 10 (a), 20 (b), and 30 (c) min of cycling at 60% heart rate reserve under normobaric hypoxic conditions (FIO_2_ ≈ 15 ± 0.1%, ~9000 ft) is plotted against skeletal muscle (*vastus lateralis*) oxygen saturation (SmO_2_). SmO_2_ is expressed as the change from rest (Δ) and quantified as the area under the curve (AUC), representing the integrated local deoxygenation response during exercise. Males are shown as circles and females as triangles. Each panel included 22 participants.

Skeletal muscle oxygenation is presented in Table 3. *Vastus lateralis* deoxygenation increased from 10 to 20 min (−30 ± 17% vs. −22 ± 21%, *p* = 0.011) and was not different between 20 and 30 min (−22 ± 21% vs. −18 ± 18%, *p* = 0.055; Table 2). Males exhibited greater deoxygenation compared with females when averaged across time (males: −32 ± 14%; females: −15 ± 18%, *p* = 0.022). SmO_2_ AUC was strongly and inversely correlated with dyspnea at 10 min (*r* = −0.561, *p* = 0.007), 20 min (*r* = −0.558, *p* = 0.007), and 30 min (*r* = −0.568, *p* = 0.006; Figure 3). When adjusted for wattage, SmO_2_ remained correlated and the interpretation was unchanged with bootstrapped confidence intervals (*r* = −0.428 to −0.523, all *p* < 0.047). Sensitivity analyses using Huber robust regression yielded consistent results, indicating that the observed associations were not driven by influential observations (all robust *p* < 0.012).

### 3.4 Microvascular responsiveness and autonomic activity in relation to dyspnea

NIRS‐VOT parameters (Table 4) and HRV indices (Table 5) did not differ between high and low dyspnea groups (all *p* > 0.147 and all *p* > 0.393, respectively). At the group level, neither NIRS‐VOT parameters (all *p* > 0.138) nor indices of HRV (all *p* > 0.326) were significantly associated with dyspnea perception during exercise.

**TABLE 4:** Vascular occlusion test parameters.

Variable	All (*n* = 22)	High dyspnea (*n* = 7)	Low dyspnea (*n* = 7)	*p*‐value	Male (*n* = 11)	Female (*n* = 11)	*p*‐value
Desaturation rate (%· s^−1^)	−0.33 ± 0.09	−0.34 ± 0.10	−0.32 ± 0.09	0.674	−0.31 ± 0.09	−0.34 ± 0.09	0.459
Nadir (%)	−61 ± 14	−60 ± 15	−59 ± 14	0.945	−56 ± 14	−66 ± 14	0.124
Resaturation rate (%· s^−1^)	3.2 ± 1.6	3.3 ± 1.2	2.3 ± 0.9	0.147	3.0 ± 1.0	3.5 ± 2.0	0.453
Maximum ∆ (%)	5 (3–7)	6 (5–11)	4 (3–7)	0.497	6 (5–11)	3 (2–4)	**0.003** ^a^
2‐min AUC (%· s)	400 (240–556)	440 (351–847)	327 (259–547)	0.722	492 (400–847)	221 (125–423)	**0.008** ^a^
Nadir to peak (∆%)	67 ± 13	68 ± 13	65 ± 11	0.716	65 ± 11	69 ± 14	0.437

*Note*: Normally distributed values are presented as skeletal muscle oxygen saturation (SmO_2_) means ± SD, and non‐parametric data are median (Q1–Q3). Nadir (%) represents the lowest SmO_2_ during occlusion, and maximum (%) represents the peak SmO_2_ during reperfusion following cuff release.

Abbreviation: AUC, area under the curve.

^a^ Statistically significant difference between males and females (*p* < 0.05). Significant *p*‐values have been bolded.

**TABLE 5:** Heart rate variability indices.

Variable	All (*n* = 22)	High dyspnea (*n* = 7)	Low dyspnea (*n* = 7)	*p*‐value	Male (*n* = 11)	Female (*n* = 11)	*p*‐value
HR (bpm)	60 ± 11	59 ± 13	57 ± 12	0.702	62 ± 11	58 ± 10	0.401
RMSSD (ms)	85.3 ± 51.8	91.5 ± 46.5	81.3 ± 56.3	0.717	59.8 ± 38.7	110.8 ± 52.0	**0.017** ^a^
SDNN (ms)	69.4 ± 34.8	74.6 ± 28.2	62.9 ± 39.0	0.530	54.6 ± 28.5	84.3 ± 35.4	**0.043** ^a^
pNN50 (%)	45.0 ± 26.7	48.5 ± 25.3	43.8 ± 28.0	0.748	30.7 ± 24.8	59.2 ± 20.9	**0.009** ^a^
LF (ms^2^)	1036 (578–2090)	1246 (764–2367)	769 (588–1036)	0.456	997 (446–1611)	1064 (754–2447)	0.270
Ln LF (ms^2^)	6.8 ± 1.1	7.1 ± 0.8	6.6 ± 1.4	0.393	6.6 ± 1.2	7.1 ± 1.0	0.215
HF (ms^2^)	1499 (827–4846)	1304 (910–4282)	1463 (975–3788)	1.000	1110 (317–1419)	3562 (1882–6412)	**0.013** ^a^
Ln HF (ms^2^)	7.3 ± 1.4	7.5 ± 1.2	7.2 ± 1.5	0.717	6.6 ± 1.4	8.1 ± 0.9	**0.008** ^a^
LF/HF	0.5 (0.4–1.2)	0.5 (0.4–1.3)	0.5 (0.4–0.9)	1.000	0.9 (0.4–1.7)	0.5 (0.2–0.6)	0.066
Ln LF/HF	−0.5 ± 1.0	−0.4 ± 0.8	−0.6 ± 0.7	0.585	−0.0 ± 0.9	−1.0 ± 0.8	**0.018** ^a^

*Note*: Parametric variables are presented as mean ± SD. Non‐parametric variables are presented as median (Q1‐Q3).

Abbreviations: HF, high‐frequency power; HR, heart rate; LF, low‐frequency power; LF/HF, ratio of low‐ to high‐frequency power; ln, natural logarithm; pNN50, percentage of successive NN intervals differing by >50 ms; RMSSD, root mean square of successive differences; SDNN, standard deviation of normal‐to‐normal intervals.

^a^ Statistically significant difference between males and females (*p* < 0.05). Significant *p*‐values have been bolded.

For NIRS‐VOT parameters (Table 4), males exhibited higher maximum values compared with females (6 [5–11] vs. 3 [2–4] %, *p* = 0.003) and greater 2‐min AUC (492 [400–847] vs. 221 [125–423] %·s, *p* = 0.008), whereas all other parameters did not differ between sexes (all *p* > 0.124). For HRV indices (Table 5), females demonstrated higher RMSSD (*p* = 0.017), SDNN (*p* = 0.043), pNN50 (*p* = 0.009), HF power (*p* = 0.013), ln HF (*p* = 0.008), and lower ln LF/HF (*p* = 0.018) compared with males. LF power, ln LF, and LF/HF did not differ significantly between sexes (all *p* > 0.066).

## 4 DISCUSSION

While acute normobaric hypoxia is known to reduce SpO_2_ (Amann et al., 2007; Hogan et al., 1999) and increase ventilatory and sympathetic responses (Somers et al., 1989a, 1989b), interindividual variability in dyspnea perception during hypoxic exercise was poorly understood. The primary findings of the present investigation under normobaric hypoxic conditions (FIO_2_ ≈ 15%, equivalent to ~9000 ft) were: (1) marked interindividual variability in dyspnea responses across the 30‐min submaximal cycling protocol; (2) microvascular responsiveness and autonomic activity were not associated with dyspnea perception but greater skeletal muscle deoxygenation was; and (3) males exhibited higher dyspnea and greater skeletal muscle deoxygenation compared to females at the same relative intensity.

### 4.1 Interindividual differences in dyspnea during hypoxic exercise

In the present study, marked interindividual variability in dyspnea responses was observed across the 30‐min submaximal cycling protocol. To facilitate comparisons between individuals with distinct dyspnea responses, participants were categorized into high and low dyspnea groups while individuals presenting in the middle were removed from the analysis. Notably, all components of the immediate perception domain, including breathing discomfort (A1) and the sensory qualities (SQ1–SQ5: physical breathing effort, air hunger, tightness, mental breathing effort, and hyperpnea), were significantly greater in the high dyspnea group compared to the low dyspnea group. This suggests that dyspnea during normobaric hypoxia is indeed a multidimensional perceptual experience, characterized by concurrent elevations across both affective and sensory dimensions.

The marked interindividual variability in dyspnea observed in the present study may have important implications for exercise tolerance and training responses under hypoxic conditions. Dyspnea is a key perceptual signal contributing to exercise limitation (Laviolette & Laveneziana, 2014), and greater dyspnea perception has been linked to earlier task termination (Amann et al., 2007). Accordingly, individuals who experience greater dyspnea at a given relative intensity may exhibit reduced exertional tolerance, which could, in turn, influence adherence to hypoxic training interventions (Laviolette & Laveneziana, 2014). From a practical perspective, this variability also suggests that prescribing exercise based solely on physiological metrics (e.g., %HRR or power output) may not reflect the perceptual load experienced by individuals when in this unique training environment. Instead, perceptual measures such as dyspnea or RPE may offer a complementary approach to individualizing exercise intensity, potentially allowing for more consistent perceptual stimuli across individuals, albeit at the expense of absolute workload (Eston, 2012; Tucker, 2009). Such an approach may have implications for optimizing both adherence and the stimulus for physiological adaptation under hypoxic conditions, though this remains an area of further exploration.

### 4.2 Physiological contributions to dyspnea during normobaric hypoxia

Contrary to our hypotheses, microvascular responsiveness and autonomic activity were not associated with dyspnea perception. While interindividual differences in microvascular responsiveness (at ~4900 and 5600 m) (Martin et al., 2013), as well as in oxygen saturation and autonomic activity (at FIO_2_ ≈ 9.6%) (Botek et al., 2015), have been reported under hypoxic conditions, these environments represent more severe hypoxia than is typically encountered in simulated altitude settings (~13%–16% FIO_2_) (Girard et al., 2020; Liu et al., 2025). The present study employed a more ecologically accessible hypoxic stimulus (FIO_2_ ≈ 15%), and it is therefore possible that this was insufficient to elicit measurable interindividual differences with the present experimental methodology. Alternatively, it is possible that microvascular responsiveness and autonomic activity are not the primary drivers of interindividual differences in dyspnea under normobaric hypoxia. For instance, previous work has shown that pharmacological reduction of sympathetic activity did not alter perceived dyspnea during exercise in healthy humans (Galloway et al., 2000), suggesting that autonomic activity may not be a primary determinant of dyspnea perception across contexts. Although interindividual variability in hypoxic ventilatory response and arterial oxygenation (Teppema & Dahan, 2010) has been observed previously, SpO_2_ did not differ between dyspnea groups in the present study. This suggests that additional physiological mechanisms beyond systemic oxygenation may also contribute to interindividual differences in dyspnea. Notably, although microvascular responsiveness was not associated with dyspnea in the present study, prior work at sea level has demonstrated robust relationships between NIRS‐derived indices of microvascular responsiveness assessed at rest and aerobic capacity (V̇O_2_max) (Koutlas et al., 2024; Lesmana et al., 2026), suggesting that its functional relevance may be context‐dependent and potentially altered under hypoxic conditions due to differences in the subjective experience of acute altitude exposure.

Interestingly, SmO_2_ was strongly and inversely correlated with dyspnea at all timepoints of cycling, and this relationship remained after adjustment for wattage. Although SmO_2_ did not differ significantly between dyspnea groups, the magnitude of the group difference was moderate‐to‐large across exercise time points (Cohen's d = 0.70–0.98), suggesting that the absence of statistical significance may reflect limited power associated with the small tertile‐based sample (*n* = 7/group), rather than absence of a physiologically meaningful difference. Together, these observations suggest that greater local skeletal muscle deoxygenation is associated with greater dyspnea perception during exercise in simulated altitude. Given that oxygen delivery represents a combination of convective (i.e., muscle blood flow) and diffusive (i.e., red blood cell desaturation) components (Boushel et al., 2014; Mendelson et al., 2021; Tucker et al., 2019), it is likely that greater local desaturation compensated, in part, for attenuated convective delivery in the context of SpO_2_ reductions. This mismatch in oxygen delivery–demand matching may have promoted the accumulation of metabolic byproducts that activate group III/IV muscle afferents, thereby augmenting ventilatory responses and contributing to dyspnea (Amann et al., 2010). Taken together, these findings suggest that while resting parameters (e.g., NIRS‐VOT and HRV) do not appear to contribute to interindividual variability in dyspnea under normobaric hypoxia, the local skeletal muscle oxygen environment may play an important role and represents an objective physiological outcome relating to the metabolic environment of the muscle that may be used when prescribing exercise training intensities in this environment.

### 4.3 Biological sex differences in dyspnea

In support of our hypothesis, males reported greater dyspnea than females. Although the overall immediate perception domain was greater in males, not all component scores differed by sex. Specifically, breathing discomfort (A1), physical breathing effort (SQ1), and tightness (SQ3) did not differ, whereas males reported higher ratings of air hunger (SQ2), mental breathing effort (SQ4), and hyperpnea (SQ5). This suggests that sex differences in dyspnea may be driven more by specific sensory qualities related to ventilatory drive and cognitive effort, rather than by the affective intensity of breathing discomfort per se. Consistent with this, the proportion of males was higher in the high dyspnea group compared to the low dyspnea group (86% vs. 29%, *p* = 0.103), though we were underpowered to detect this difference after group partitioning.

In the present study, males exhibited greater deoxygenation at the vastus lateralis compared to females (*p* = 0.022). This suggests that the local skeletal muscle oxygen environment may contribute to the observed sex differences in dyspnea, though this remains to be confirmed as correlation does not suggest causation. Although absolute cycling intensity did not significantly differ between sexes (*p* = 0.110), males consistently exercised at numerically higher workloads across time points (Table 3), which may have contributed to the greater specific sensory dyspnea qualities observed in males despite matched relative intensity. Moreover, sex differences in autonomic activity and microvascular responsiveness were observed in the present study, consistent with previous literature in healthy populations studied under normoxic conditions. However, neither NIRS‐VOT parameters (all *p* > 0.138) nor indices of HRV (all *p* > 0.326) were associated with dyspnea perception, suggesting that these physiological differences may not represent primary determinants of sex differences in dyspnea under normobaric hypoxia. For instance, males exhibited a greater 2‐min AUC (Table 4), suggesting a greater sustained microvascular reperfusion response, consistent with evidence reported under normoxic conditions (Citherlet et al., 2024; Rasica et al., 2022). Additionally, females exhibited greater HRV indices, including RMSSD, SDNN, pNN50, and HF power (Table 5), indicating greater parasympathetic modulation at rest, consistent with meta‐analytic evidence in healthy humans studied under normoxic conditions (Koenig & Thayer, 2016). Thus, the local skeletal muscle oxygen environment during exercise, rather than resting autonomic activity and microvascular responsiveness, may help explain the observed sex differences in dyspnea to acute normobaric exercise.

Previous work performed under normoxic conditions has generally reported little or no biological sex differences in dyspnea during submaximal exercise in young healthy adults, with differences becoming more evident during maximal exercise or in older populations (Cory et al., 2015; Molgat‐Seon et al., 2018). In contrast, the present study demonstrated greater dyspnea in males during acute normobaric hypoxia, suggesting that the influence of sex on dyspnea perception may differ between normoxic and hypoxic exercise conditions. Further studies are warranted to clarify these potential differences as the present study performed exercise at a fixed relative intensity not an absolute oxygen demand.

### 4.4 Experimental considerations

We recognize that while the subjective experience of dyspnea may differ between people, we used the Multidimensional Dyspnea Profile as a robust tool to measure dyspnea as it has been extensively validated while demonstrating strong test–retest reliability, construct validity, and responsiveness (Banzett et al., 2015). While exercise intensity can influence dyspnea, the present cycling exercise was completed at the same relative intensity. The absence of differences in RPE and SmO_2_ between the 20‐ and 30‐min time points suggests that a steady state was achieved and provides confidence in the precision of the relative exercise intensity titration across participants. While HRV is a marker of autonomic nervous system activity, indices may be influenced by breathing frequency via respiratory sinus arrhythmia; however, paced breathing was not implemented to preserve spontaneous ventilatory patterns. The present study did not assess leg dominance, and NIRS placement was standardized to the right vastus lateralis across participants. Despite potential differences in NIRS placement relative to leg dominance, leg dominance has been shown to not affect SmO_2_ during cycling (Skotzke et al., 2024). Further, NIRS‐VOT parameters have also been shown to be unaffected by limb dominance (Bezemer et al., 2009). In addition, NIRS‐VOT does not directly measure microvascular blood flow, capillary hemodynamics, or vascular conductance. Rather, it serves as a surrogate of vascular responsiveness by exploring NIRS‐derived local oxygenation during the post‐occlusive reactive hyperemia period. Although cycling heart strain differed between dyspnea groups, absolute values remained well below the manufacturer‐defined threshold for moderate strain (<0.1 mV). Furthermore, the chest strap‐derived metric reflects generalized ST‐segment deviation rather than specific elevation or depression, limiting physiological interpretation. As such, the significance of this finding remains unclear. The present study was conducted under a fixed hypoxic stimulus (FIO_2_ ≈ 15 ± 0.1%) in healthy, altitude‐naïve individuals, so the results may not generalize to populations with different characteristics or training environments. Carbon dioxide concentration was not measured in the present environment, but continuous air exchange maintains ambient carbon dioxide levels in relation to atmospheric air. Future work should examine the physiological contributors to interindividual variability in dyspnea across a range of hypoxic intensities within the moderate domain in different populations.

## 5 CONCLUSION

The growing accessibility of simulated altitude facilities (typically ~13%–16% FIO_2_) has expanded their use to broader recreational and non‐athletic populations. Under normobaric hypoxic conditions (FIO_2_ ≈ 15%, equivalent to ~9000 ft), the present study demonstrates marked interindividual variability in dyspnea responses during submaximal cycling at a standardized relative intensity. While microvascular responsiveness and autonomic nervous system activity were not associated with dyspnea perception, greater skeletal muscle deoxygenation was. Additionally, males exhibited higher dyspnea and greater skeletal muscle deoxygenation compared to females at the same relative intensity. These findings highlight the importance of interindividual variability in dyspnea during hypoxic exercise and warrant further investigation into its functional and training‐related implications.

## AUTHOR CONTRIBUTIONS

**Jacob L. Schwartz:** Conceptualization; data curation; formal analysis; investigation; methodology; project administration; validation; visualization. **Aisha Khan:** Data curation; formal analysis; investigation; project administration. **Robert F. Bentley:** Conceptualization; formal analysis; funding acquisition; methodology; resources; supervision; validation.

## FUNDING INFORMATION

This project was supported by Natural Sciences and Engineering Research Council (NSERC) of Canada Discovery Grant RGPIN‐2022‐04443 (to R.F.B.). J.L.S. was supported by an Ontario Graduate Scholarship. A.K. was supported by a NSERC Undergraduate Student Research Award.

## CONFLICT OF INTEREST STATEMENT

The authors declare no conflicts of interest.

## ETHICS STATEMENT

The University of Toronto Health Sciences research ethics board approved this study (Approval No. 47308) according to the terms of the Declaration of Helsinki and all participants provided written informed consent before their participation.

## Data Availability

The data that support the findings of this study are available from the corresponding author upon reasonable request.
